# Economic burden of non-alcoholic steatohepatitis with significant fibrosis in Thailand

**DOI:** 10.1186/s12876-021-01720-w

**Published:** 2021-03-25

**Authors:** Pochamana Phisalprapa, Ratthanon Prasitwarachot, Chayanis Kositamongkol, Pranaidej Hengswat, Weerachai Srivanichakorn, Chaiwat Washirasaksiri, Sombat Treeprasertsuk, Phunchai Charatcharoenwitthaya, Nathorn Chaiyakunapruk

**Affiliations:** 1 Division of Ambulatory Medicine, Department of Medicine, Faculty of Medicine Siriraj Hospital, Mahidol University, Bangkok, Thailand; 2 Department of Pharmacy Technicians, Sirindhorn College of Public Health Suphanburi, Suphanburi, Thailand; 3 Faculty of Medicine Siriraj Hospital, Mahidol University, Bangkok, Thailand; 4 Division of Gastroenterology, Department of Medicine, Faculty of Medicine, Chulalongkorn University, and King Chulalongkorn Memorial Hospital, Thai Red Cross, Bangkok, Thailand; 5 Division of Gastroenterology, Department of Medicine, Faculty of Medicine Siriraj Hospital, Mahidol University, Bangkok, Thailand; 6 Department of Pharmacotherapy, College of Pharmacy, University of Utah, Salt Lake City, UT 84112 USA

**Keywords:** Non-alcoholic steatohepatitis, NASH, Significant fibrosis, Economic burden, Cost of illness, Prevalence, Thailand

## Abstract

**Background:**

Non-alcoholic steatohepatitis (NASH) has been recognised as a significant form of chronic liver disease and a common cause of cirrhosis and hepatocellular carcinoma, resulting in a considerable financial burden on healthcare resources. Currently, there is no information regarding the economic burden of NASH in low- and middle-income countries (LMICs). The aim of this study was to estimate the economic burden of NASH in Thailand as a lesson learned for LMICs.

**Methods:**

To estimate the healthcare costs and prevalence of NASH with significant fibrosis (fibrosis stage ≥ 2) in the general Thai population, an eleven-state lifetime horizon Markov model with 1-year cycle length was performed. The model comprised Thai population aged 18 years and older. The cohort size was based on Thailand Official Statistic Registration Systems. The incidence of NASH, transitional probabilities, and costs-of-illness were based on previously published literature, including systematic reviews and meta-analyses. The age-specific prevalence of NASH was based on Thai NASH registry data. Costs were expressed in 2019 US Dollars ($). As we undertook analysis from the payer perspective, only direct medical costs were included. All future costs were discounted at an annual rate of 3%. A series of sensitivity analyses were performed.

**Results:**

The estimated total number of patients with significant NASH was 2.9 million cases in 2019, based on a NASH prevalence of 5.74%. The total lifetime cost of significant NASH was $15.2 billion ($5,147 per case), representing approximately 3% of the 2019 GDP of Thailand. The probabilistic sensitivity analysis showed that the lifetime costs of significant NASH varied from $11.4 billion to $18.2 billion.

**Conclusions:**

The economic burden associated with NASH is substantial in Thailand. This prompts clinicians and policy makers to consider strategies for NASH prevention and management.

**Supplementary Information:**

The online version contains supplementary material available at 10.1186/s12876-021-01720-w.

## Background

The excess accumulation of fat in the liver in patients without other causes such as excessive alcohol consumption or viral hepatitis is recognised as non-alcoholic fatty liver disease (NAFLD) [1]. It is a spectrum of diseases comprising two distinct conditions: non-alcoholic fatty liver (NAFL) and non-alcoholic steatohepatitis (NASH) [2]. The latter is a severe form in which the steatosis is accompanied by lobular inflammation and hepatocyte ballooning that can progress to liver fibrosis, compensated cirrhosis, decompensated cirrhosis, hepatocellular carcinoma (HCC), and other liver-related and non-liver related mortalities such as cardiovascular diseases [3–7]. These are the causes of the mounting number of hospital admissions, which are likely to continue to increase every year.

NASH is now considered the second most common indication for liver transplantation in the United States after chronic hepatitis C [8]. A number of previous studies have revealed that patients with NASH are at high risk for advanced liver disease [9–11], and those with significant fibrosis have a significantly higher risk of overall mortality than the general population [12]. A study on the epidemiology and disease burden of non-alcoholic steatohepatitis suggested that NASH can affect 3% to 5% of the global population, with minor variations at the country-specific level [13]. A study focusing on the economic impact of NASH in Hong Kong suggested that the projected cost of NASH over 20 years would be $1.32 billion, at $257 per-person year [14], while a study on the lifetime cost of advanced NASH in the United States demonstrated that the condition costs a total of $96.18 billion, at $139,724 per patient [15].

In Thailand, several studies have calculated the incidence and prevalence of NAFLD. Previous studies showed that the prevalence of NAFLD varied from 24.58 to 67% depending on patients’ underlying diseases [16–19]. However, information concerning the cost of illness of NASH—particularly NASH with significant fibrosis—is still limited in Thailand as well as in other low- and middle-income countries (LMICs). Certainly, an understanding of the economic burden of NASH in Thailand might be useful in helping policy makers with the development of strategies to manage this disease, which is likely to become a significant health issue in the near future. Therefore, this study set out to estimate the economic burden of NASH with significant fibrosis (fibrosis stage ≥ 2), using Thailand as an example of an LMIC.

## Methods

### Description of model

**Fig. 1:**
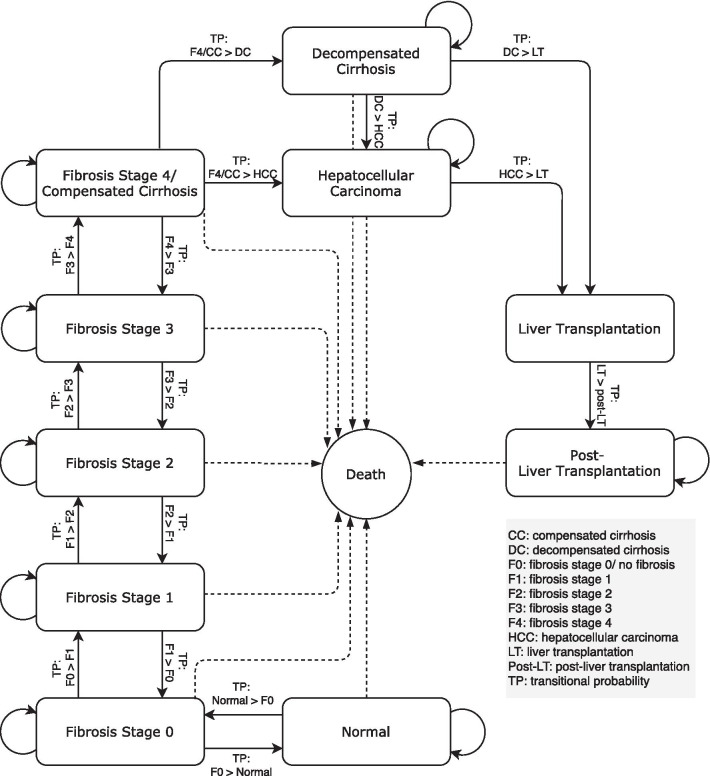
A Markov model of non-alcoholic fatty liver disease. CC compensated cirrhosis, DC decompensated cirrhosis, F0 fibrosis stage 0/ no fibrosis, F1 fibrosis stage 1, F2 fibrosis stage 2, F3 fibrosis stage 3, F4 fibrosis stage 4, HCC hepatocellular carcinoma, LT liver transplantation, Post-LT post-liver transplantation, TP transitional probability

A Markov model, which was built in Microsoft Excel, was adapted from a study conducted by Chongmelaxme et al*.* [20] to estimate the health care costs and prevalence of NASH with significant fibrosis (fibrosis stage 2 and higher) in Thailand. The model consisted of the normal state and the ten other health states, namely, no fibrosis (F0), fibrosis stage 1 (F1), fibrosis stage 2 (F2), fibrosis stage 3 (F3), compensated cirrhosis or fibrosis stage 4 (F4), decompensated cirrhosis, HCC, liver transplantation (LT), post-liver transplantation (post-LT), and death. The model was developed adhering to the natural history of the disease, which progressively worsens to more severe states, and regression was allowed only from F0 to normal, F1 to F0, F2 to F1, F3 to F2, and F4 to F3 (Fig. 1). The model comprised the general Thai population aged 18 years and older (51 million people) at the beginning of the model. In the analysis, the population in each age cohort entered the model at the normal state, and in the next cycle, they either remained in this state or transited to fibrosis stage 0 or death. The annual cycle length and a lifetime horizon were used in the model. The size of the population of each age group was based on Thailand Official Statistic Registration Systems, and the age-specific mortality rate (ASMR) was based on Global Health Observatory data from the World Health Organization [21, 22] (Additional file 1: Appendix 1 and 2). This model was performed from a payer perspective. All costs were discounted at the rate of 3%, in accordance with the recommendations of the Thai Health Technology Assessment (HTA) guidelines [23].

### Input parameters

Overall, the input parameters were based on a literature review and country-specific data sources. All input parameters are detailed in (Table 1). The prevalence of NAFLD in the general Thai population was pooled from previously published local studies [17–19, 24], and the age-specific prevalence of NASH with significant fibrosis was calculated from the NASH registry data of Siriraj Hospital, Mahidol University, and previous publications [25, 26] (see Additional file 1: Appendix 3 and 4). The transitional probabilities were mainly based on the study of Chongmelaxme et al*.* [20], which was the previous, model-based, economic evaluation study in Thailand.

**Table 1:** Input parameters, values, and data sources

Parameters	Base-case values	SE	References
*Prevalence (NAFLD) in general population*			
Age 18–39	0.353	0.013	[17–19, 24]
Age 40–59	0.348	0.002	[17–19, 24]
Age 60 +	0.244	0.004	[17–19, 24]
*Prevalence (NASH with significant fibrosis) in NAFLD*			
Age 18–29	0.183	0.009	[25, 26]
Age 30–39	0.224	0.011	[25, 26]
Age 40–49	0.169	0.009	[25, 26]
Age 50–59	0.164	0.008	[25, 26]
Age 60–69	0.152	0.008	[25, 26]
Age 70–79	0.090	0.005	[25, 26]
Age 80 +	0.090	0.005	[25, 26]
*Transitional probabilities*			
Progression			
Normal > F0			[15]
Age 18–39	0.00140		
Age 40–59	0.00407		
Age 60–89	0.00515		
Age 90 +	0.00150		
F0 > F1	0.090	0.026	[27]
F1 > F2	0.140	0.036	[27]
F2 > F3	0.070	0.020	[27]
F3 > F4/CC	0.080	0.020	[27]
F4 > DC	0.060	0.010	[27]
F4 > HCC	0.030	0.012	[28, 29]
DC > HCC	0.030	0.01173	[29–32]
DC > LT	0.0003	0.00002	[33–35]
HCC > LT	0.0016	0.00008	[33, 34]
HCC > Death	0.4490	0.02959	[22, 37]
LT > post-LT	0.7993	NA	
LT > Death	0.2007	0.01024	[38, 44]
Post-LT > Death	0.0653	0.00333	[38, 44]
*Regression*			
F0 > normal	0.000		Assumption
F1 > F0	0.080	0.026	[27]
F2 > F1	0.190	0.059	[27]
F3 > F2	0.200	0.087	[27]
F4 > F3	0.120	0.023	[27]
*Direct medical costs (2019 $)*			
NASH	89	11	[40]
CC	2121	271	[41]
DC	3916	499	[41]
HCC	4787	622	[41]
LT	18,703	2298	[41]
post-LT	2908	371	[41]

CC, compensated cirrhosis; DC, decompensated cirrhosis; F0, fibrosis stage 0/ no fibrosis; F1, fibrosis stage 1; F2, fibrosis stage 2; F3, fibrosis stage 3; F4, fibrosis stage 4; HCC, hepatocellular carcinoma; LT, liver transplantation; NAFLD, non-alcoholic fatty liver disease; NASH, non-alcoholic steatohepatitis; post-LT, post-liver transplantation; SE, standard error

### Transitional probabilities

The transitional probabilities for all fibrosis stages were reanalysed using data from a systematic review and meta-analysis estimating the rates of fibrosis progression [27]. The transitional probabilities from F4 to decompensated cirrhosis and to HCC were based on published articles [28–32], whereas that of patients experiencing liver transplantation was derived from country-specific studies [33–35]. The age-specific mortality rate (ASMR) of the Thai population [22] multiplied by the relative risks of mortality among NAFLD and cirrhosis patients [36] was used as the mortality rate. The mortality rate of HCC patients was derived from a study estimating the mortality rate of Thai patients with HCC [37]. Since data on the mortality rate of transplanted NASH patients are not available, this study applied the death rate associated with liver transplants and post-liver transplantation in chronic hepatitis B and C patients [38].

### Costs

Since this study was conducted from a payer perspective, only the direct medical costs were estimated. A micro-costing method was utilised to estimate the cost of health care utilisation. The outpatient costs included outpatient medication care and laboratory testing, with NAFLD patients being assumed to visit an outpatient clinic four times a year [39]. The relevant costs were obtained from a costing database in Thailand [40]. The treatment costs only related to NAFLD and NASH. As the costs for compensated cirrhosis, decompensated cirrhosis, and HCC are not available, they were assumed to be the same as for hepatitis C patients and were adapted from a country-specific study [41]. However, the costs of hepatitis C medications and viral testing were excluded. Also, it can be assumed that the costs of management of DC, HCC, LT, and post-LT would be the same in patients with advanced liver disease, regardless of the disease etiologies [15]. All costs were adjusted using the Consumer Price Index and reported in 2019 US Dollars (1 US Dollar = 32.3 Thai Baht) [42, 43].

### Analysis

The outcome measures were the first-year cost, the fifth-year cost and the lifetime cost for each age group. The prevalence and incidence of NAFLD and the NASH with significant fibrosis population were estimated for 2019. A probabilistic sensitivity analysis (PSA) was performed to assess uncertainty and the impact of the base-case input parameters on the model outputs and their robustness. The sensitivity analyses were conducted for all age groups.

## Results

### Prevalence of NAFLD and NASH with significant fibrosis

The model estimated that the total NAFLD prevalence rate would be 32.17% and the total NASH with significant fibrosis prevalence rate would be 5.74% in 2019. It was found that the 18–39 age group would have the highest prevalence rate, with 35.12% and 7.14% for NAFLD and NASH with significant fibrosis, respectively. In 2019, of the total NAFLD cohort, 17.84% of the cases were estimated to have NASH. Based on the Thailand population, our model predicted that the total number of adult patients with NAFLD would be 16.6 million, while the number of NASH patients with significant fibrosis would be 2.9 million in 2019. Additionally, about 50% of the NASH with significant fibrosis population in 2019 would comprise adults aged under 39 (Table 2; Additional file 1: Appendix 5).

**Table 2:** Prevalence of NAFLD and NASH with significant fibrosis in Thailand in 2019

Age group	Thai population size	NAFLD prevalence (%)	NAFLD cohort	NASH with significant fibrosis prevalence (%)	NASH with significant fibrosis cohort
18–39	20,533,776	35.12	7,212,381	7.14	1,466,099
40–59	19,919,412	34.49	6,869,359	5.80	1,155,851
60 +	11,136,059	22.57	2,513,210	3.05	339,981
Total	51,589,247	32.17	16,594,951	5.74	2,961,931

NAFLD, non-alcoholic fatty liver disease; NASH, non-alcoholic steatohepatitis

### Estimated costs

We estimated the economic burden of NASH with significant fibrosis at the first-year, fifth year and over a lifetime. In the base-case analysis, the first-year costs would be $886,664,483, the fifth-year costs would be $886,376,246, and the total lifetime cost for NASH with significant fibrosis was projected to be $15.2 billion or $5,174 per case. In 2019, the GDP of Thailand was $543 billion which this burden account for approximately 3% of the 2019 GDP. On one hand, we found that the highest estimated lifetime cost per case would be for adults aged over 60, mounting to $9,492 per patient, while the lowest cost per case would be in the 18–39 age cohort, at $4,393 per case. In contrast, the lifetime cost of the population aged 18–39 would account for 42% of the total amount, whereas that of patients aged over 60 would account for 21% of the total (Table 3; Additional file 1: Appendix 6).

**Table 3:** Lifetime cost for NASH with significant fibrosis

Age group	Total cases	1st–year cost ($)	5th-year cost ($)	Lifetime cost ($)	Lifetime cost per case ($)
18–39	1,466,099	214,323,428	283,001,011	6,440,244,930	4393
40–59	1,155,851	240,136,567	330,194,687	5,577,909,108	4826
60 +	339,981	432,204,487	273,180,548	3,227,197,465	9492
Total	2,961,931	886,664,483	886,376,246	15,245,351,503	5147

All costs reported in 2019 US Dollars

### Sensitivity analysis

**Fig. 2:**
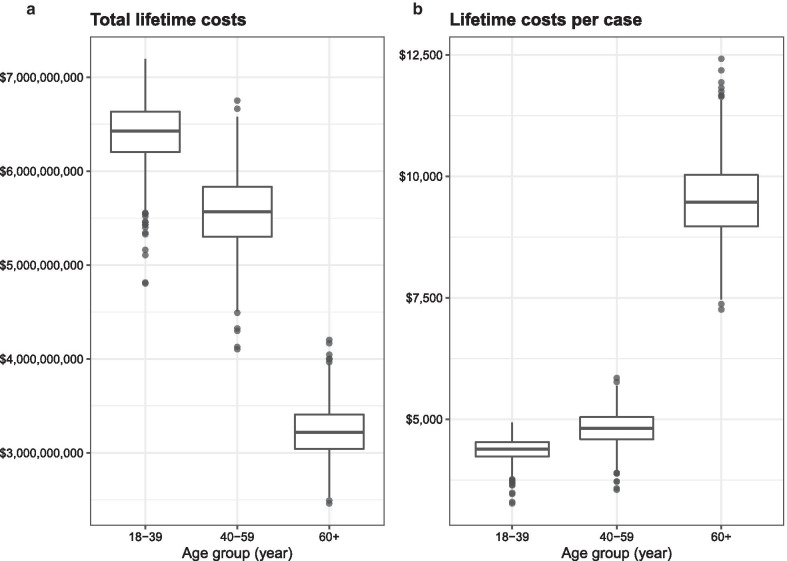
Probabilistic sensitivity analysis (PSA). PSA represented by box plots showing the median, 25th percentile, 75th percentile, and outlier cost of NASH with significant fibrosis of each age group (2019 $) (**a**) total lifetime costs (**b**) lifetime cost per case

Probabilistic sensitivity analyses were conducted for the NASH with significant fibrosis patients and included all age groups (Additional file 1: Appendix 7). The results showed that the total cases varied from 2.94 million to 2.99 million in 2019. Moreover, the lifetime cost of NASH with significant fibrosis was found to be around $15.2 billion, varying from $11.4 billion to $18.2 billion, with the lifetime cost per case averaging $5,147, ranging from $3,833 to $6,150 (Table 4; Fig. 2a, b).

**Table 4:** Sensitivity analysis results

Age group	Total cases (Min–Max)	Lifetime cost ($) (Min–Max)	Lifetime cost per case ($) (Min–Max)
18–39	1,466,099 (1,445,077–1,486,251)	6,440,244,930 (4,804,286,359–7,196,682,390)	4393 (3266–4934)
40–59	1,155,851 (1,141,140–1,168,039)	5,577,909,108 (4,103,739,487–6,751,636,344)	4826 (3549–5849)
60 +	339,981 (336,251–343,547)	3,227,197,465 (2,461,384,957–4,203,115,813)	9492 (7259–12,418)
Total	2,961,931 (2,937,772–2,986,282)	15,245,351,503 (11,369,410,803–18,151,434,547)	5147 (3833–6150)

All costs reported in 2019 US Dollars

## Discussion

This study showed that the estimated prevalence of NASH with significant fibrosis in the adult Thai population in 2019 would be 2.9 million cases, with an estimated cost of $886,376,245 in the fifth year. The predicted lifetime cost of this liver condition would be $15.2 billion, or $5,174 per case, which represented approximately 3% of the 2019 GDP of Thailand [45].

The NAFLD prevalence of Thailand from our study is relatively higher than those for Africa, North America, Europe, and other Asian countries, but similar to those for South America and the Middle East [9]. As to NASH, the total estimated NASH prevalence rate of our study, 5.74% of the Thai population, is similar to the rates for Canada and the US (5.2% [46] and 5.3% [47], respectively). However, these rates are slightly higher than the global prevalence, which ranges between 3 and 5% [13]. Interestingly, compared with other Asian countries such as China (2.4% [47]), Japan (3.0% [47]) and Hong Kong (4.5% [14]), the estimated NASH prevalence rate for Thailand is higher. This higher prevalence may be influenced by differences in ethnicity, genetic composition, diets, and sedentary lifestyles [14, 48]. Also, previous data showed that the growth rate of overweight and obesity has been much higher in recent decades in Thailand as compared to those countries [49]. However, these contributing factors need to be explored further. Nevertheless, awareness of this high prevalence for Thailand is important because it could urge clinicians and policy makers to focus on, and prepare for, the significant burdens presented by this disease.

In addition, when considered individual age groups, our results revealed that the lower-aged population has a higher prevalence of NAFLD and NASH. This could result from the older population having lower levels of fat deposits in the liver [50–52], which might be explained by the accumulated fat in the liver being burned out and becoming more fibrotic. Moreover, younger people today will face a higher burden of metabolic syndrome as compared to earlier generations [53], this could contribute to higher NAFLD and NASH prevalence in the lower-aged cohort.

The total lifetime cost of NASH is substantially high, being equivalent to approximately 3% of the GDP of Thailand for 2019 [45]. Focusing on each age group, we found that the lifetime cost per case for the younger age group was lower than that for the older group. Although the life expectancy of the older population is shorter than that of the younger population, the total lifetime cost per case is double that of the older population because more advanced diseases and higher progression rates are detected in the older population. Additionally, our results reveal the significant burden from NASH with significant fibrosis which is not only comparable with other significant health issues in Thailand such as alcohol consumption [54], smoking-related diseases [55], and obesity [56], but also higher in terms of economic burden resulting in the loss of health care resources and the country’s economy.

Evidently, NASH with significant fibrosis cause the financial burden in both developing and developed countries. When comparing the total number of NASH cases and the financial burden revealed by our study with the corresponding data from developed countries [15], the costs and the number of cases in Thailand are lower. Nevertheless, they have a relatively high impact when considered in relation to the Thai economy.

It is important to note that our study estimated the burden from a payer perspective; hence, the costs of comorbidities, nonmedical costs, and societal costs of NASH were excluded from the model. This means that the economic burden of this disease is likely to have been markedly underestimated. If such parameters were included, the total costs of NASH would be larger because it would include the money spent by patients and their household members, as well as non-monetary losses, such as opportunity costs and lost income caused by illness.

In Thailand's context, health care services are still restricted, particularly human resources. This is a critical issue that must be considered. There is a significant human impact both in terms of the healthcare professionals needed to deal with the disease and its consequences, and the loss of productive workers due to the illness. From our findings, around 3 million cases of NASH are predicted in 2019, yet there are only about 61,000 clinicians in Thailand [57]. This equates to a ratio of 1:50 for the doctors available to the number of NASH with significant fibrosis patients. There is an even lower ratio for the current number of hepatologists in Thailand (1:6,000 for NASH with significant fibrosis, and 1:130,000 for the general population, of whom about 6% may be diagnosed with NASH at any time). These ratios indicate that if a strategy is not developed to prevent NASH and its consequences, there will be insufficient numbers of medical professionals to provide adequate care to all patients and to effectively monitor the disease. As well, there will not be sufficient funding available to support patients from low-income households or those receiving healthcare via Thailand's Universal Coverage Scheme.

Thus, we should develop strategies to prevent NASH and its progressive state; otherwise, we will have to deal with the high costs of the disease. Screening is one possible strategy. It could enable early disease detection and promote early treatment, which would require relatively low funding. However, the cost of the screening strategy itself should be considered; therefore, a further study on screening costs is required.

Both NASH and its complications present huge burdens. For example, while cirrhosis and HCC need high-cost treatments, these diseases typically have poor outcomes. This study only considered NASH and its consequent diseases; when we take the real-world situation into account, there is much evidence that NASH is associated with comorbidities such as diabetes, obesity, dyslipidaemia, metabolic syndrome and cardiovascular diseases [8, 14, 58–60]. It may also lead to higher mortality and a higher financial burden for those with underlying diseases [10, 36]. Thus, predictions of the economic and financial burdens of the disease are important evidence in support of the need for preventative strategies, especially in developing countries.

To our knowledge, this is the first study to report the economic burden of NASH in LMICs. This study used Thailand as an example LMIC to reflect the increased burden of NASH in Southeast Asia. Our results are consistent with other studies that claimed that the burden of NAFLD and NASH are large and are ever increasing [14, 15, 46, 59]. The findings of the present study highlight that the prevention and treatment of NASH should be addressed by every LMIC in the world.

A key strength of this study was that its findings are likely to be valid as most of the input data used for the model were drawn from local sources. Secondly, the probabilistic sensitivity analysis that we conducted determined that the model was robust in its estimations of the costs of NASH with significant fibrosis.

There are some limitations to this study. First, there is a lack of biopsy-proven NAFLD prevalence data for the Thai general population. We therefore pooled the prevalence of NAFLD from studies [17–19, 24] that had diagnosed the disease using imaging tests (ultrasonography) and transient elastography techniques (FibroScan). Because of their higher sensitivity, these information sources provided a more accurate disease prevalence than studies that had used liver function tests as the diagnostic method. Moreover, the prevalence of NAFLD was consistent with the previous systematic review and meta-analysis in Asia [61]. Second, there are no direct assessments of the incidence and prevalence of NASH in NAFLD patients, in view of the high risk of severe complications from liver biopsies conducted on NAFLD patients without any explicit risk of having NASH with significant fibrosis. Consequently, the NASH with significant fibrosis data were limited. Hence, we adopted the biopsy-proven and elastography-confirmed NASH with significant fibrosis prevalence to signify the proportion of NAFLD cases in Thailand [25, 26] as well as to minimise errors from the sampling variability that may occur in biopsy diagnostic method [62]. Third, we used the constant rate of liver transplantation which does not vary according to age of the patients. Nevertheless, the probability of liver transplantation was based on local data [33–35]. Forth, due to the unavailability of local or NASH-specific data, a few input parameters were retrieved from international studies, for instance, the transitional probabilities of fibrosis progression and regression. As well, the mortality rates of liver transplant and post-transplantation patients were adopted from the rates of Thai patients with hepatitis B and hepatitis C infections. Nevertheless, this information should be able to be used as representative data for the Thai population [20]. Fifth, regardless of clinical manifestation, entire patients in this model who had been diagnosed NASH were assumed to receive standard treatment and applied in the cost calculation. In the real-world practice, those without clinical abnormality might not receive the treatment. Lastly, our study did not consider other non-liver-related events which are known to associated with NASH such as cardiovascular diseases, obesity, etc. Thus, the total costs will increase if the treatment of those comorbidities are included.

## Conclusions

NASH with significant fibrosis cause substantial economic burden in Thailand. This should prompt clinicians and policy makers to pay more attention in developing and implementing the effective strategies for the prevention and management of NASH.

## Supplementary Information

**Additional file 1: Appendix 1**. Thai population; **Appendix 2**. Age-specific mortality rate of Thai general population, NAFLD patients and cirrhosis patients; **Appendix 3**. Prevalence of non-alcoholic fatty liver disease (NAFLD) in Thailand; **Appendix 4**. Prevalence of non-alcoholic steatohepatitis with significant fibrosis in Thailand; **Appendix 5**. Estimated number of patients in advanced health state; **Appendix 6**. The proportion of total costs by health state; **Appendix 7**. Probabilistic sensitivity analysis.

## Data Availability

The datasets analysed during the current study are available from the corresponding author on reasonable request.

## Abbreviations

ASMR: Age specific mortality rate
CC: Compensated cirrhosis
F0: Fibrosis stage 0/ no fibrosis
F1: Fibrosis stage 1
F2: Fibrosis stage 2
F3: Fibrosis stage 3
F4: Fibrosis stage 4
HCC: Hepatocellular carcinoma
HTA: Health Technology Assessment
LMICs: Low- and middle-income countries
LT: Liver transplantation
NAFLD: Non-alcoholic fatty liver disease
NAFL: Non-alcoholic fatty liver
NASH: Non-alcoholic steatohepatitis
post-LT: Post-liver transplantation
