# Supplementary material for: Economic burden of non-alcoholic steatohepatitis with significant fibrosis in Thailand
Source: BMC Gastroenterol. 2021 Mar 25;21:135. doi: 10.1186/s12876-021-01720-w (PMC7992785; doi:10.1186/s12876-021-01720-w)
Supplement: Supplementary file 1 — Additional file 1: Appendix 1. Thai population; Appendix 2. Age-specific mortality rate of Thai general population, NAFLD patients and cirrhosis patients; Appendix 3. Prevalence of non-alcoholic fatty liver disease (NAFLD) in Thailand; Appendix 4. Prevalence of non-alcoholic steatohepatitis with significant fibrosis in Thailand; Appendix 5. Estimated number of patients in advanced health state; Appendix 6. The proportion of total costs by health state; Appendix 7. Probabilistic sensitivity analysis. [file 12876_2021_1720_MOESM1_ESM.pdf]

ELECTRONIC SUPPLEMENTARY MATERIAL

Economic Burden of Non-alcoholic Steatohepatitis with Significant Fibrosis in Thailand

Pochamana Phisalprapa, M.D., Ph.D.[1],
Ratthanon Prasitwarachot, M.Sc.[2],
Chayanis Kositamongkol, Pharm.D.[1],
Pranaidej Hengswat, B.Ec.[3],
Weerachai Srivanichakorn, M.D.[1],
Chaiwat Washirasaksiri, M.D., M.Sc.[1],
Sombat Treeprasertsuk, M.D, Ph.D.[4],
Phunchai Charatcharoenwitthaya, M.D.[5],
Nathorn Chaiyakunapruk, Pharm.D., Ph.D.[6, *]

[1]Division of Ambulatory Medicine, Department of Medicine, Faculty of Medicine Siriraj Hospital, Mahidol University, Bangkok, Thailand
[2]Department of Pharmacy Technicians, Sirindhorn College of Public Health Suphanburi, Suphanburi, Thailand
[3]Faculty of Medicine Siriraj Hospital, Mahidol University, Bangkok, Thailand
[4]Division of Gastroenterology, Department of Medicine, Faculty of Medicine, Chulalongkorn University, and King Chulalongkorn Memorial Hospital, Thai Red Cross, Bangkok, Thailand
[5]Division of Gastroenterology, Department of Medicine, Faculty of Medicine Siriraj Hospital, Mahidol University, Bangkok, Thailand
[6]Department of Pharmacotherapy, College of Pharmacy, University of Utah, Salt Lake City, UT, USA

***Corresponding Author**
Professor Nathorn Chaiyakunapruk Pharm.D., Ph.D.
Department of Pharmacotherapy, College of Pharmacy, University of Utah, Salt Lake City, UT, USA, 84112
Tel: (801) 585-3092; E-mail: nathorn.chaiyakunapruk@utah.edu

Table of Contents

Appendix 1: Thai population **......3**

Appendix 2: Age-specific mortality rate of Thai general population, NAFLD patients and cirrhosis patients **......5**

Appendix 3: Prevalence of non-alcoholic fatty liver disease (NAFLD) in Thailand **......9**

Appendix 4: Prevalence of non-alcoholic steatohepatitis with significant fibrosis in Thailand **......17**

Appendix 5: Estimated number of patients in advanced health state **......19**

Appendix 6: The proportion of total costs by health state **......21**

Appendix 7: Probabilistic sensitivity analysis **......22**

References **......24**

Appendix 1: Thai population

Table S1 the number of Thai population, according to age and gender, and the total number of each age cohort [1]

Age	Male	Female	Total	Total per age group
18	407,410	387,539	794,949	
19	428,108	409,373	837,481	
20	428,100	407,591	835,691	
21	449,438	436,784	886,222	
22	490,461	472,693	963,154	
23	497,807	482,834	980,641	
24	492,692	478,916	971,608	
25	477,384	462,048	939,432	
26	479,967	466,748	946,715	
27	487,328	474,480	961,808	
28	484,343	471,187	955,530	
29	476,896	466,389	943,285	
30	457,432	447,178	904,610	
31	447,350	438,088	885,438	
32	443,513	438,683	882,196	
33	458,528	453,769	912,297	
34	475,856	471,953	947,809	
35	477,725	475,699	953,424	
36	487,940	488,590	976,530	
37	504,394	506,744	1,011,138	
38	504,213	507,131	1,011,344	
39	514,384	518,090	1,032,474	20,533,776
40	513,723	521,065	1,034,788	
41	494,954	506,364	1,001,318	
42	518,230	532,860	1,051,090	
43	509,887	528,727	1,038,614	
44	498,965	520,056	1,019,021	
45	505,658	531,351	1,037,009	
46	492,874	521,850	1,014,724	
47	505,670	540,850	1,046,520	
48	510,221	551,547	1,061,768	
49	498,865	538,998	1,037,863	
50	487,720	533,642	1,021,362	
51	502,465	552,837	1,055,302	
52	479,479	526,837	1,006,316	
53	464,854	518,559	983,413	
54	469,474	524,592	994,066	
55	463,717	523,829	987,546	
56	435,160	487,993	923,153	
57	423,220	474,424	897,644	
58	401,832	453,711	855,543	
59	396,472	455,880	852,352	19,919,412
60	378,141	434,683	812,824	

**Age**	**Male**	**Female**	**Total**	**Total per age group**
61	343,769	401,080	744,849	
62	329,635	386,042	715,677	
63	302,950	352,396	655,346	
64	279,663	329,940	609,603	
65	282,467	340,058	622,525	
66	259,791	314,332	574,123	
67	253,375	306,427	559,802	
68	235,748	287,611	523,359	
69	221,683	273,663	495,346	
70	211,843	264,598	476,441	
71	187,295	232,597	419,892	
72	167,531	209,796	377,327	
73	150,727	191,750	342,477	
74	132,398	171,077	303,475	
75	125,640	161,102	286,742	
76	114,638	150,711	265,349	
77	107,104	143,320	250,424	
78	103,923	142,025	245,948	
79	88,761	124,883	213,644	
80	88,192	124,616	212,808	
81	82,047	118,092	200,139	
82	71,178	106,586	177,764	
83	63,712	96,679	160,391	
84	55,882	87,654	143,536	
85	48,757	75,923	124,680	
86	42,656	69,113	111,769	
87	37,354	60,627	97,981	
88	29,752	50,663	80,415	
89	24,791	42,492	67,283	
90	20,387	35,637	56,024	
91	16,973	29,477	46,450	
92	13,834	24,363	38,197	
93	10,176	17,928	28,104	
94	8,105	14,156	22,261	
95	5,953	9,935	15,888	
96	4,987	7,834	12,821	
97	3,947	6,134	10,081	
98	3,366	4,960	8,326	
99	2,932	4,067	6,999	
≥ 100	8,234	10,735	18,969	11,136,059
**Total**	**24,865,006**	**26,724,241**	**51,589,247**	**51,589,247**

Appendix 2: Age-specific mortality rate of Thai general population, NAFLD patients and cirrhosis patients

Age-specific mortality rate (ASMR) of Thai general population was based on WHO life table 2016 [2]. ASMRs of NAFLD and cirrhosis patients were calculated by multiplying hazard ratio by ASMR of general population, as shown in the equations below.

ASMR of NAFLD patients = 1.29 × ASMR of general population

ASMR of cirrhosis patients = 3.13 × ASMR of general population

The hazard ratios of NAFLD and cirrhosis for overall death were based on the study of Ekstedt *et al*. [3]. This cohort study was conducted in Sweden and was intended to determine the disease-specific mortality in NAFLD patients and to evaluate the NAFLD Activity score (NAS) and fibrosis stage as prognostic markers for overall and disease-specific mortality. All 229 participants were confirmed with NAFLD by liver biopsy and had been followed-up for up to 33 years. The authors reported that the hazard ratio of NAFLD for overall mortality was 1.29 (95% CI: 1.04-1.59, p = 0.020), while that for cirrhosis was 3.13 (95% CI: 1.08-9.12, p = 0.036).

The ASMRs of NAFLD and cirrhosis of the Thai adult population aged 18 to 100 are shown in Table S2.

Table S2 Age-specific mortality rates and probability of deaths of Thai population, NAFLD patients and cirrhosis patients

Age (years)	ASMR of general population	Probability of death of general population	ASMR of NAFLD	Probability of death of NAFLD	ASMR of cirrhosis	Probability of death of cirrhosis
18	0.0010	0.0010	0.0013	0.0013	0.0032	0.0032
19	0.0010	0.0010	0.0013	0.0013	0.0032	0.0032
20	0.0015	0.0015	0.0020	0.0019	0.0047	0.0047
21	0.0015	0.0015	0.0019	0.0019	0.0047	0.0047
22	0.0015	0.0015	0.0019	0.0019	0.0047	0.0047
23	0.0015	0.0015	0.0019	0.0019	0.0047	0.0047
24	0.0015	0.0015	0.0019	0.0019	0.0047	0.0047
25	0.0020	0.0020	0.0026	0.0026	0.0063	0.0063
26	0.0020	0.0020	0.0026	0.0026	0.0063	0.0063

Age (years)	ASMR of general population	Probability of death of general population	ASMR of NAFLD	Probability of death of NAFLD	ASMR of cirrhosis	Probability of death of cirrhosis
27	0.0020	0.0020	0.0026	0.0026	0.0063	0.0063
28	0.0020	0.0020	0.0026	0.0026	0.0063	0.0063
29	0.0020	0.0020	0.0026	0.0026	0.0063	0.0063
30	0.0025	0.0025	0.0032	0.0032	0.0079	0.0078
31	0.0025	0.0025	0.0032	0.0032	0.0079	0.0078
32	0.0025	0.0025	0.0032	0.0032	0.0079	0.0078
33	0.0025	0.0025	0.0032	0.0032	0.0078	0.0078
34	0.0025	0.0025	0.0032	0.0032	0.0078	0.0078
35	0.0025	0.0025	0.0032	0.0032	0.0078	0.0078
36	0.0025	0.0025	0.0032	0.0032	0.0078	0.0078
37	0.0025	0.0025	0.0032	0.0032	0.0078	0.0078
38	0.0025	0.0025	0.0032	0.0032	0.0078	0.0078
39	0.0025	0.0025	0.0032	0.0032	0.0078	0.0078
40	0.0035	0.0035	0.0045	0.0045	0.0109	0.0109
41	0.0035	0.0035	0.0045	0.0045	0.0109	0.0108
42	0.0035	0.0035	0.0045	0.0045	0.0109	0.0108
43	0.0035	0.0035	0.0045	0.0045	0.0109	0.0108
44	0.0035	0.0035	0.0045	0.0045	0.0109	0.0108
45	0.0045	0.0045	0.0058	0.0057	0.0140	0.0139
46	0.0045	0.0044	0.0057	0.0057	0.0140	0.0139
47	0.0044	0.0044	0.0057	0.0057	0.0139	0.0138
48	0.0044	0.0044	0.0057	0.0057	0.0139	0.0138
49	0.0044	0.0044	0.0057	0.0057	0.0139	0.0138
50	0.0059	0.0059	0.0076	0.0076	0.0185	0.0183
51	0.0059	0.0059	0.0076	0.0076	0.0185	0.0183
52	0.0059	0.0059	0.0076	0.0076	0.0185	0.0183
53	0.0059	0.0059	0.0076	0.0076	0.0184	0.0183
54	0.0059	0.0059	0.0076	0.0076	0.0184	0.0183
55	0.0083	0.0083	0.0108	0.0107	0.0261	0.0258

**Age (years)**	**ASMR of general population**	**Probability of death of general population**	**ASMR of NAFLD**	**Probability of death of NAFLD**	**ASMR of cirrhosis**	**Probability of death of cirrhosis**
56	0.0084	0.0083	0.0108	0.0107	0.0262	0.0258
57	0.0084	0.0083	0.0108	0.0107	0.0262	0.0258
58	0.0083	0.0083	0.0108	0.0107	0.0261	0.0258
59	0.0083	0.0083	0.0107	0.0107	0.0261	0.0257
60	0.0118	0.0117	0.0152	0.0151	0.0369	0.0362
61	0.0118	0.0117	0.0152	0.0151	0.0368	0.0362
62	0.0118	0.0117	0.0152	0.0151	0.0368	0.0362
63	0.0118	0.0117	0.0152	0.0151	0.0369	0.0362
64	0.0118	0.0117	0.0152	0.0150	0.0368	0.0361
65	0.0181	0.0179	0.0233	0.0231	0.0566	0.0550
66	0.0181	0.0179	0.0233	0.0230	0.0566	0.0550
67	0.0181	0.0179	0.0233	0.0230	0.0566	0.0550
68	0.0181	0.0179	0.0233	0.0230	0.0565	0.0549
69	0.0180	0.0179	0.0233	0.0230	0.0564	0.0549
70	0.0283	0.0279	0.0366	0.0359	0.0887	0.0849
71	0.0284	0.0280	0.0366	0.0359	0.0887	0.0849
72	0.0283	0.0279	0.0365	0.0359	0.0887	0.0848
73	0.0283	0.0279	0.0365	0.0358	0.0885	0.0847
74	0.0282	0.0278	0.0364	0.0358	0.0884	0.0846
75	0.0444	0.0435	0.0573	0.0557	0.1391	0.1299
76	0.0443	0.0434	0.0572	0.0556	0.1388	0.1296
77	0.0443	0.0433	0.0571	0.0555	0.1386	0.1294
78	0.0442	0.0432	0.0570	0.0554	0.1383	0.1292
79	0.0441	0.0431	0.0568	0.0553	0.1379	0.1288
80	0.0743	0.0716	0.0958	0.0914	0.2325	0.2075
81	0.0742	0.0715	0.0957	0.0913	0.2322	0.2072
82	0.0740	0.0713	0.0955	0.0911	0.2316	0.2068
83	0.0739	0.0713	0.0954	0.0910	0.2314	0.2066
84	0.0738	0.0711	0.0952	0.0908	0.2310	0.2062

Age (years)	ASMR of general population	Probability of death of general population	ASMR of NAFLD	Probability of death of NAFLD	ASMR of cirrhosis	Probability of death of cirrhosis
85	0.1502	0.1395	0.1938	0.1762	0.4702	0.3751
86	0.1500	0.1393	0.1935	0.1759	0.4695	0.3747
87	0.1500	0.1393	0.1935	0.1759	0.4695	0.3747
88	0.1498	0.1391	0.1932	0.1757	0.4688	0.3742
89	0.1497	0.1391	0.1932	0.1757	0.4687	0.3742
90	0.1496	0.1390	0.1930	0.1755	0.4684	0.3740
91	0.1497	0.1390	0.1931	0.1756	0.4685	0.3740
92	0.1496	0.1390	0.1930	0.1755	0.4683	0.3739
93	0.1496	0.1390	0.1930	0.1755	0.4683	0.3739
94	0.1496	0.1390	0.1930	0.1756	0.4684	0.3740
95	0.1499	0.1392	0.1933	0.1758	0.4691	0.3744
96	0.1502	0.1394	0.1937	0.1761	0.4700	0.3750
97	0.1502	0.1395	0.1938	0.1762	0.4702	0.3751
98	0.1505	0.1397	0.1941	0.1765	0.4710	0.3756
99	0.1508	0.1400	0.1945	0.1768	0.4720	0.3762
≥ 100[a]	0.1511	1.0000	0.1949	1.0000	0.4730	1.0000

ASMR, age-specific mortality rate; NAFLD, non-alcoholic fatty liver disease

[a]We assumed that no one lives longer than 100 years.

Appendix 3: Prevalence of non-alcoholic fatty liver disease (NAFLD) in Thailand

Characteristics of studies included for calculating the prevalence of NAFLD in Thailand

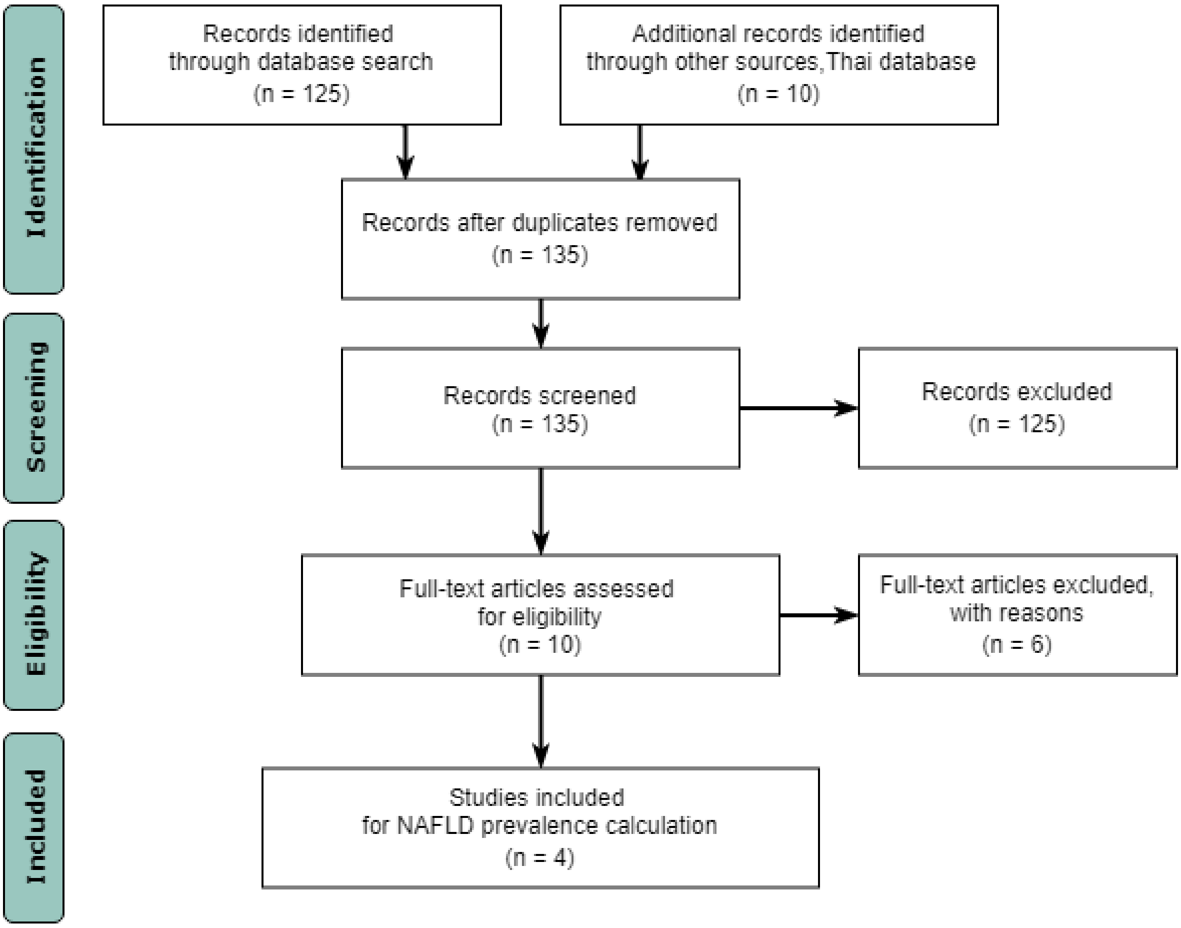

Figure S1 PRISMA diagram of included and excluded studies for calculating the prevalence of NAFLD in Thailand
NAFLD, non-alcoholic fatty liver disease

To identify the prevalence of non-alcoholic fatty liver in Thailand, a literature search was performed in PubMed. The search terms “prevalence”, “NAFLD”, “nonalcoholic fatty liver disease”, “Thailand” and “Thai” were used to identify relevant articles published in peer-reviewed journals published before 31 January 2020. One hundred and twenty-five articles were found. In order to collect more information, a search using search terms in the Thai language was also performed in Google Scholar, Thai Journal Online, and ThaiLIS; ten articles were found. After the duplicates were removed and the remaining articles were screened, ten articles were eligible for full-text assessment (Fig S1).

Udompornmongkol *et al.* [4] conducted a study at Detudom Royal Crown Prince Hospital, Ubon Ratchathani, Northeastern Thailand, on 300 participants with an average age of 52.35 years and an average BMI of 24.78 kg/mg^2. They reported that the prevalence of ultrasound-diagnosed non-alcoholic fatty liver disease (NAFLD) was 46%.

A study by Vanduangden *et al.* [5] was conducted at King Chulalongkorn Memorial Hospital on 161 subjects. All were examined with controlled attenuation parameter with transient elastography (CAP-TE). The study revealed that 99 out of 161 participants (61.5%) had NAFLD.

Ekpanyapong *et al.* [6] used CAP-TE to evaluate the prevalence and risk factors of NAFLD and liver fibrosis in 180 type 2 diabetic patients with normal serum aminotransferase. The results showed that the prevalence of NAFLD was 82.8%.

Another study aimed to identify the prevalence of NAFLD and significant hepatic fibrosis in diabetic patients. Conducted by Sobhonslidsuk *et al.* [7], the study investigated 137 diabetic patients identified with NAFLD by CAP-TE. The prevalence of NAFLD from this study was 60.16%.

A study entitled “Prevalence and Risk Factors of Non-Alcoholic Fatty Liver Disease in Asian Individuals with Metabolic Syndrome” by Phisalprapa *et al.* [8] investigated the prevalence of NAFLD in 509 patients. The research revealed that, overall, 67% were diagnosed with NAFLD; of these, 30% had a mild fatty liver, and 37% had a moderate to severe fatty liver.

Nevertheless, we believe that the prevalence of NAFLD increases with age, and this was considered to affect the accuracy of our analysis. Consequently, studies by Udompornmongkol *et al.*, Vanduangden *et al.*, Ekpanyapong *et al.*, Sobhonslidsuk *et al.*, and Phisalprapa *et al.* [4-8] were excluded since those studies did not report the age-specific NAFLD prevalences. Additionally, a study by Summart *et al.* [9] , which reported a prevalence of 21.85% for ultrasound-diagnosed NAFLD among 34,709 participants, was also excluded

since the population of this study was the same population as the study conducted by Thinkhamrop *et al*. [10], which was already included.

Hence, of the 10 studies, four met the inclusion criteria and were utilised for the meta-analysis to determine the pooled prevalence of NAFLD in the Thai population. The characteristics of the included studies are presented in Table S3.

Table S3 Characteristics of studies included in the meta-analysis for pooled prevalence of NAFLD in the general Thai population

Characteristic	Author, publication year			
	**Pitug *et al*., 2017** [11]	**Leelaprasart *et al*., 2015** [12]	**Thinkhamrop *et al*., 2015** [10]	**Rattanangamkul *et al*., 2017** [13]
Setting (hospital, province)	Borabue Hospital, Maha Sarakham	Krabi Hospital, Krabi	CASCAP (9 tertiary care hospitals)	Phayathai Hospital, Bangkok
Region	Northeast	South	Northeast	Central
Study design	cross-sectional	cross-sectional (retrospective review)	population-based, prospective cohort, survey study	cross-sectional (retrospective review)
Sample size	329	720	45,263	4,471
Characteristics of participants				
Age (years)[a]	55.5±8.6	52.64±16.42	53.46±9.25	45
Male (%)	34.65%	41.67%	42.3%	48.32%
BMI (kg/m^2)[b]	24.09	NA	NA	24.77
Overweight/ Obesity[c]	60.79%	27.08%	NA	10.46%
With comorbidities	23.10%	HT: 19.44%<br>T2DM: 15.83%<br>DLP: 16.53%<br>Central obesity: 22.08%	CCA: 35.1%<br>Smoking: 23.1%<br>Alcohol consumption[d]: 43.8%<br>HB: 2%	15.95%<br>(T2DM, HT, DLP, others)

**Characteristic**	**Author, publication year**			
	**Pitug *et al*., 2017** [11]	**Leelaprasart *et al*., 2015** [12]	**Thinkhamrop *et al*., 2015** [10]	**Rattanangamkul *et al*., 2017** [13]
			HC: 0.2%	
			HT: 2.9%	
NAFLD diagnosis method	ultrasound	ultrasound	ultrasound	ultrasound
NAFLD prevalence, regarding age groups				
20-39 years	0/0	31/173	0/0	498/1,271
		(17.92%)		(39.18%)
40-49 years	29/91		3,516/17,756	926/2,042
	(31.87%)	96/316	(19.80%)	(45.35%)
50-59 years	58/137	(30.38%)	3,901/16,204	790/1,458
	(42.34%)		(24.07%)	(54.18%)
≥ 60 years	37/101	50/231	2,227/11,303	0/0
	(36.63%)	(21.65%)	(19.70%)	
Overall	124/329 (37.69%)	177/720	9,644/45,263	2,214/4,771
		(24.58%)	(21.30%)	(46.41%)

BMI, body mass index; CCA, cholangiocarcinoma; CASCAP, the Cholangiocarcinoma Screening and Care Program; DLP, dyslipidaemia; HB, hepatitis B infection; HC, hepatitis C infection; HT, hypertension; NA, not applicable; NAFLD, non-alcoholic fatty liver disease; T2DM, type 2 diabetes mellitus

[a]When the average age of the cohort was not reported, it was assumed by using the sum of the median age multiplied by the number of participants

in each age group, divided by the total number of participants, as shown in the equation below. If the maximum limit of the age range was unknown, the minimum age, regarding age range, would be used instead of median age.

$$Average\ age = \frac{\sum(median\ age\ \times number\ of\ participant\ in\ each\ age\ group)}{total\ number\ of\ participants}$$

[b]Average BMI was also assumed using the same method as average age when it was not reported.

[c]Cutoff value of BMI for indicating overweight/obesity was different in each study. Pitug *et al*. [11] used BMI ≥ 23 kg/m^2 to indicate being overweight. Leelaprasart *et al*. [12] used BMI ≥ 27 kg/m^2 and ≥ 25 kg/m^2 to indicate obesity in men and women, respectively. Rattanangamkul *et al*. [13] used BMI cut-off at 30 kg/m^2 to indicate obesity.

[d]CASCAP did not report the amount of alcohol consumption of the participants. Although the effects of alcohol consumption on non-alcoholic fatty liver disease remain controversial, according to the crude analysis of this study, the author reported that alcohol was neither a protective nor a risk factor. In addition, a study by Tajima *et al*. suggested that the association of alcohol consumption with fatty liver prevalence was non-linear [14]. These may imply that alcohol intake has less effect on fatty liver prevalence. Therefore, we included the NAFLD prevalence from this study in the meta-analysis.

Result of pooled prevalence of NAFLD in Thailand

The pooled prevalence of NAFLD was pooled using a random-effects model by Stata Statistical Software: Release 14 (StataCorp LP, College Station, TX, USA).

Table S4 Pooled prevalence of NAFLD in Thailand, classified by age group

Age group (years)	NAFLD prevalence	95% confidence interval
20-39	35.34%	32.91%–37.77%
40-59	34.84%	17.13%–52.56%
≥ 60	24.43%	17.39%–31.50%

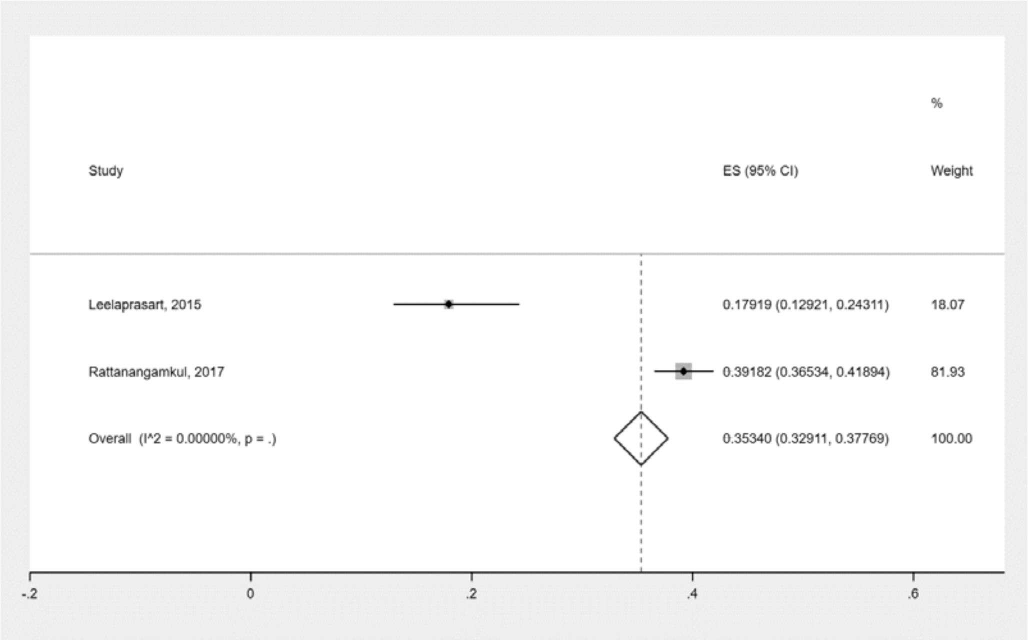

Figure S2 Prevalence of NAFLD in Thai general population aged 20–39 years

ES, effect size; NAFLD, non-alcoholic fatty liver disease

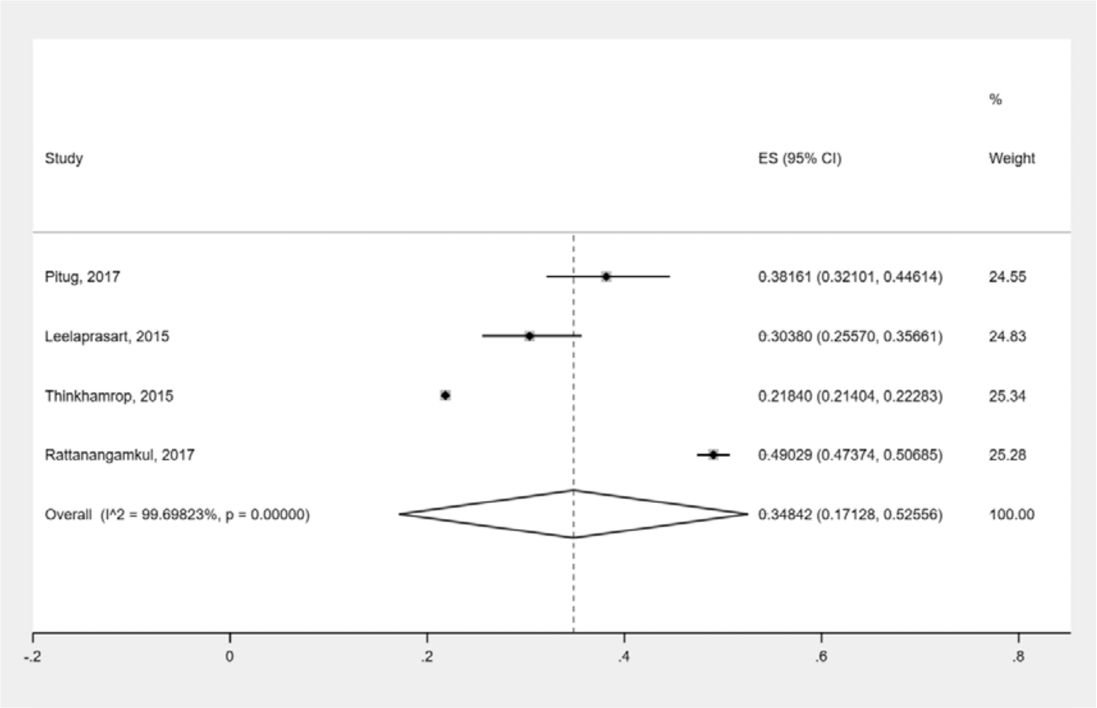

Figure S3 Prevalence of NAFLD in Thai general population aged 40–59 years
ES, effect size; NAFLD, non-alcoholic fatty liver disease

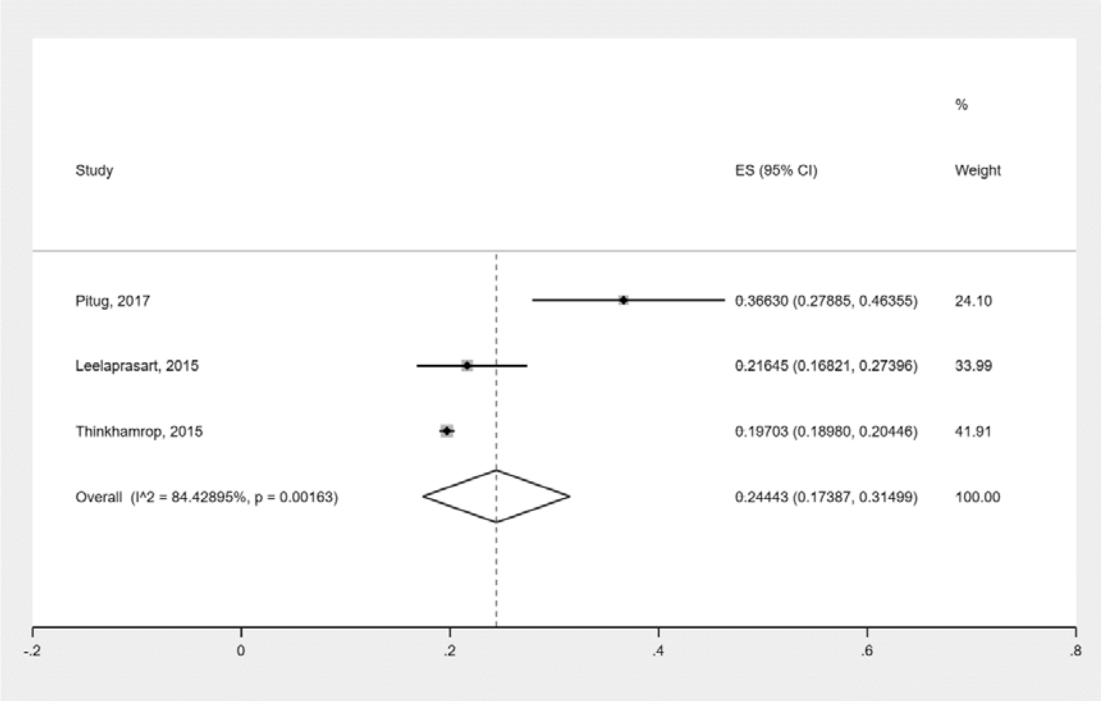

Figure S4 Prevalence of NAFLD in Thai general population aged 60 years and over
ES, effect size; NAFLD, non-alcoholic fatty liver disease

Appendix 4: Prevalence of non-alcoholic steatohepatitis with significant fibrosis in Thailand

The prevalences of non-alcoholic steatohepatitis with significant fibrosis and first detected fibrosis stages among NAFLD patients in Thailand were limited. Thus, we had to calculate them based on the original data of two published cohort studies conducted on Thai patients.

First, the study conducted by Treeprasertsuk *et al.* [15] investigated the accuracy of noninvasive scoring systems by assessing advanced liver fibrosis in Thai adult patients with NAFLD. All patients (139 in this article and 160 additional cases) underwent a liver biopsy to define their histological liver fibrosis stages. Although most of the participants were obese, which means they are unable to represent the normal population, some had a lower BMI. Consequently, we assumed that the patients whose BMI was less than 23 kg/m^2 were considered normal population.

Second, the cohort study of Saokaew *et al.* [16]—which aimed to develop a simple risk scoring method to predict NAFLD in patients with metabolic syndrome—is of interest. It was conducted on 509 Thai patients. We obtained additional raw data from the research team of the participants who were later diagnosed with transient elastography (FibroScan); we classified the patients into their proper fibrosis stages using the cutoff values according to the study of Lee *et al.* [17].

From those two studies, the original data were pooled and calculated as the prevalence of NASH and the first detected fibrosis stages among NAFLD, as shown in Table S5, before being applied in the economic burden analysis.

Table S5 Prevalence of non-alcoholic steatohepatitis with significant fibrosis and first detected fibrosis stages among non-alcoholic fatty liver patients, classified by age group

Age group (years)	Prevalence (standard error)					
	F0	F1	F2	F3	F4/CC	NASH with significant fibrosis
18-29	0.500 (0.065)	0.317 (0.060)	0.133 (0.044)	0.050 (0.003)	0.000 (0.000)	0.183 (0.009)
30-39	0.539 (0.057)	0.237 (0.049)	0.171 (0.043)	0.039 (0.002)	0.013 (0.001)	0.224 (0.011)
40-49	0.551 (0.053)	0.281 (0.048)	0.101 (0.032)	0.056 (0.003)	0.011 (0.001)	0.169 (0.009)
50-59	0.653 (0.032)	0.183 (0.026)	0.082 (0.019)	0.073 (0.018)	0.009 (0.000)	0.164 (0.008)
60-69	0.770 (0.030)	0.079 (0.019)	0.042 (0.014)	0.016 (0.001)	0.094 (0.021)	0.152 (0.008)
≥ 70	0.850 (0.036)	0.060 (0.024)	0.030 (0.002)	0.010 (0.001)	0.050 (0.003)	0.090 (0.005)

F0, fibrosis stage 0; F1, fibrosis stage 1; F2, fibrosis stage 2; F3, fibrosis stage 3; F4/CC, fibrosis stage 4/compensated cirrhosis; HCC, hepatocellular carcinoma; LT, liver transplantation; post-LT, post liver transplantation NASH, non-alcoholic steatohepatitis

Appendix 5: Estimated number of patients in advanced health state

Table S6 Estimated number of patients in advanced health states for each year in cohort simulation

Age	Estimated number of patients			
	F4/CC	DC	New HCC	LT
**18**	0	0	0	0
**19**	319357	0	0	0
**20**	308079	19150	0	0
**21**	295405	35730	9814	21
**22**	280653	50109	9932	34
**23**	264083	62469	9921	44
**24**	246268	72939	9795	50
**25**	227812	81617	9574	54
**26**	209251	88599	9281	57
**27**	191027	94000	8934	59
**28**	173459	97931	8550	60
**29**	156765	100548	8141	61
**30**	141093	101986	7718	60
**31**	126523	102374	7292	60
**32**	113092	101847	6866	58
**33**	100790	100518	6448	57
**34**	89574	98533	6039	55
**35**	79407	96006	5643	53
**36**	70229	93031	5262	51
**37**	61980	89700	4898	49
**38**	54589	86081	4550	46
**39**	47988	82290	4220	44
**40**	42108	78371	3908	41
**41**	36883	74367	3614	39
**42**	32253	70321	3337	37
**43**	28156	66264	3077	34
**44**	24536	62252	2832	32
**45**	21346	58302	2604	30
**46**	18540	54436	2389	28
**47**	16077	50672	2189	26
**48**	13919	47023	2002	24
**49**	12030	43503	1828	22
**50**	10381	40124	1666	21
**51**	8943	36896	1515	19
**52**	7693	33826	1375	17
**53**	6606	30915	1245	16
**54**	5664	28164	1126	14
**55**	4847	25571	1015	13
**56**	4141	23141	912	12
**57**	3531	20873	818	11
**58**	3005	18757	732	10
**59**	2552	16790	653	9
**60**	2162	14965	580	8
**61**	1827	13285	514	7
**62**	1540	11743	453	6
**63**	1295	10330	398	5

Age	Estimated number of patients			
	F4/CC	DC	New HCC	LT
**64**	1086	9040	349	5
**65**	907	7864	304	4
**66**	755	6805	263	4
**67**	627	5856	227	3
**68**	518	5006	194	3
**69**	427	4249	166	2
**70**	350	3575	140	2
**71**	286	2987	118	2
**72**	232	2476	98	1
**73**	188	2033	81	1
**74**	151	1650	67	1
**75**	121	1319	54	1
**76**	96	1044	43	1
**77**	76	816	34	1
**78**	59	628	27	0
**79**	46	472	21	0
**80**	35	344	16	0
**81**	27	247	11	0
**82**	20	174	8	0
**83**	15	119	6	0
**84**	11	77	4	0
**85**	8	47	3	0
**86**	6	28	2	0
**87**	4	17	1	0
**88**	3	10	1	0
**89**	2	6	0	0
**90**	1	4	0	0
**91**	1	2	0	0
**92**	1	1	0	0
**93**	0	1	0	0
**94**	0	1	0	0
**95**	0	0	0	0
**96**	0	0	0	0
**97**	0	0	0	0
**98**	0	0	0	0
**99**	0	0	0	0
**100**	0	0	0	0

DC, decompensated cirrhosis; F4/CC, fibrosis stage 4/compensated cirrhosis; HCC, hepatocellular carcinoma; LT, liver transplantation

Appendix 6: The proportion of total costs by health state

Table S7 The proportion of total costs by heath state

Health states	Average cases per year[a]	Average cost per case ($) [b]	Total cost per year (number of cases × average cost per case) ($) [b]	Discounted lifetime cost[c] ($) [b]
NASH with F2	204,674	89	18,215,986	888,400,651
NASH with F3	122,299	89	10,884,611	516,034,475
NASH with F4	65,649	2,121	139,241,529	6,352,128,109
DC	45,536	3,916	178,318,976	6,105,564,085
HCC	7,244	4,873	35,300,012	1,346,338,807
LT	26	18,018	468,468	16,106,449
post-LT	280	2,908	814,240	20,778,926
Total	445,708	-	383,243,822	15,245,351,503

DC, decompensated cirrhosis; F2, fibrosis stage 2; F3, fibrosis stage 3; F4, fibrosis stage 4; HCC, hepatocellular carcinoma; LT, liver transplantation; post-LT, post liver transplantation

[a]Average cases per year in population age 18-77 years (life expectancy of Thai population = 77 years [18])
[b]All cost presented in 2019 US Dollars
[c]Considered the dynamic of the disease and applied 3% annual discount rate

Appendix 7: Probabilistic sensitivity analysis

We performed a 2-step multivariate probabilistic sensitivity analysis (PSA) with 1,000 Monte Carlo simulations, each, by using Microsoft Excel 2019 (Microsoft Corp, Redmond, WA, USA) in order to explore the uncertainties of parameters and to examine the robustness of our model [19].

The first PSA was performed by varying all variables simultaneously, based on the appropriate distribution assigned to each variable, to obtain the minimum and maximum values of each variable. The transitional probabilities were assigned beta distribution. All cost data were assigned gamma distribution. Hazard ratios were assigned log-normal distribution [20]. Then, clinically significant variables were selected, based on expert opinion, to undergo the latter step of PSA. Eight variables were selected: 1) prevalence of NAFLD, 2) incidence of NAFLD, 3) incidence of NASH with significant fibrosis, 4) prevalence of first detected fibrosis stage among NAFLD patients, 5) probability of death of HCC, 6) hazard ratios of mortality rate of NAFLD, 7) hazard ratios of mortality rate of cirrhosis patients, and 8) cost of NASH with significant fibrosis.

The second PSA was performed using the ranges (maximum–minimum) obtained from the first PSA. Only the 8 selected variables were varied, while the others were fixed. Then, the minimum and maximum economic burdens for each age group were acquired, as shown in Figures S4. The upper and lower ends of the lines signify the minimum and maximum costs, while the upper and lower edges of the boxes represent the 25th and 75th percentiles of the cost ranges. The middle horizontal lines in the boxes are the median costs and the filled circles are the outliers.

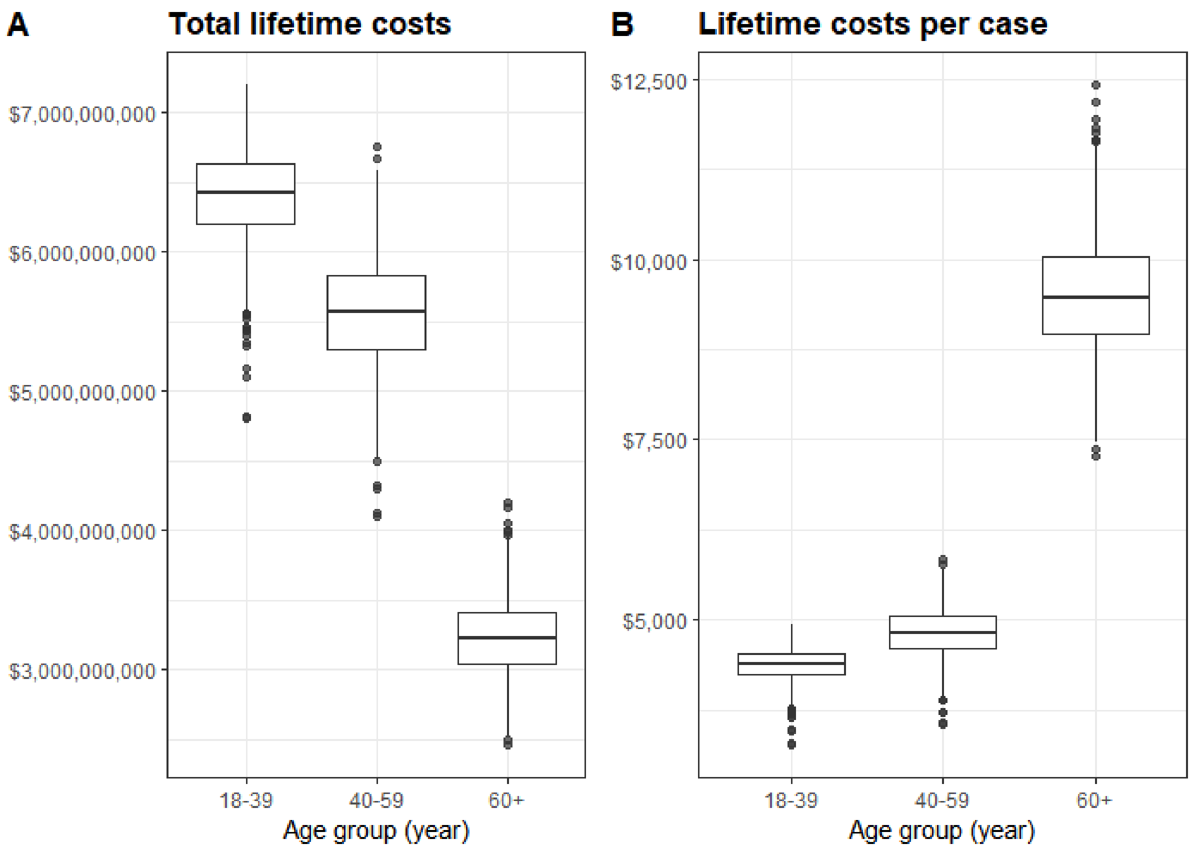

Figure S5 Probabilistic sensitivity analysis (PSA) represented by box plots showing the median, 25th percentile, 75th percentile, and outlier cost ($) of NASH with significant fibrosis of each age group (A) total lifetime costs (B) lifetime costs per case

References

1. Official statistics registration systems. Population Statistics. https://stat.bora.dopa.go.th/download/list.php. Accessed 6 Dec 2019.
2. World Health Organization. Life tables:Thailand. https://apps.who.int/gho/data/node.country.country-THA?lang-en. Accessed 20 Dec 2019.
3. Ekstedt M, Hagström H, Nasr P, Fredrikson M, Stål P, Kechagias S, Hultcrantz R. Fibrosis stage is the strongest predictor for disease-specific mortality in NAFLD after up to 33 years of follow-up. Hepatology. 2015; 61(5):1547-54.
4. Udompornmongkol C. The factors that associated with non-alcoholic fatty liver in patient undergo screening ultrasound in Detudom Royal Crown Prince Hospital, Detudom district, Ubon Rachathani province. Journal of Medicine and Public Health, Ubon Ratchathani University. 2019; 2(3):182-92.
5. Vanduangden K. Prevalence of nonalcoholic fatty liver disease (NAFLD) diagnosed by controlled attenuation parameter with transient elastography in subjects with and without metabolic syndrome. Chula Med J. 2017; 61(4):483-95.
6. Ekpanyapong S, Bunchorntavakul C, Techasirioangkun T, Napartivaumnuay N. Prevalence and risk factors of non-alcoholic fatty liver disease in type 2 diabetes mellitus patients with normal serum aminotransferase levels. J Med Assoc Thai. 2018; 101(4):S93-S103.
7. Sobhonslidsuk A, Pulsombat A, Kaewdoung P, Petraksa S. Non-alcoholic fatty liver disease (NAFLD) and significant hepatic fibrosis defined by non-invasive assessment in patients with type 2 diabetes. Asian Pac J Cancer Prev. 2015; 16(5):1789-94.
8. Phisalprapa P, Ujjin A, Apisarnthanarak P, Charoensak A, Washirasaksiri C, Srivanichakorn W, Pandejpong D, Charatcharoenwitthaya P. Sa1050 Prevalence and Risk Factors of Non-Alcoholic Fatty Liver Disease in Asian Individuals With Metabolic Syndrome. Gastroenterology. 2014; 146(5):S-947.
9. Summart U, Thinkhamrop B, Chamadol N, Khuntikeo N, Songthamwat M, Kim CS. Gender differences in the prevalence of nonalcoholic fatty liver disease in the Northeast of Thailand: A population-based cross-sectional study. F1000Res. 2017; 6.
10. Thinkhamrop K, Khuntikeo N, Phonjitt P, Chamadol N, Thinkhamrop B, Moore MA, Promthet S. Association between diabetes mellitus and fatty liver based on ultrasonography screening in the world's highest cholangiocarcinoma incidence region, Northeast Thailand. Asian Pac J Cancer Prev. 2015; 16(9):3931-6.
11. Pitug B. Prevalence of nonalcoholic fatty liver disease (NAFLD) and correlation between revalence of NAFLD and obesity in people living in borabue district , Mahasarakham province. Mahasarakham hospital journal. 2017; 14(2):22-8.
12. Leelaprasert S. Prevalence and risk factor of Non alcoholic fatty Liver Disease using abdominal ultrasound in Krabi hospital. Reg 11 Med J. 2015; 29(4):597-606.
13. Rattanangamkul M. The relation of non alcoholic fatty liver and metabolic syndrome in Thais. Durakij Pundit University; 2017.
14. Tajima R, Imamura F, Kimura T, Kobayashi S, Masuda K, Iida K. Association of alcohol consumption with prevalence of fatty liver after adjustment for dietary patterns: Cross-sectional analysis of Japanese middle-aged adults. Clin Nutr. 2020; 39(5):1580-6.
15. Treeprasertsuk S, Piyachaturawat P, Soontornmanokul T, Wisedopas-Klaikaew N, Komolmit P, Tangkijavanich P. Accuracy of noninvasive scoring systems to assess advanced liver fibrosis in Thai patients with nonalcoholic fatty liver disease. Asian Biomed (Res Rev News). 2016; 10(s1):s49-s55.

16. Saokaew S, Kanchanasuwan S, Apisarnthanarak P, Charoensak A, Charatcharoenwitthaya P, Phisalprapa P, Chaiyakunapruk N. Clinical risk scoring for predicting non-alcoholic fatty liver disease in metabolic syndrome patients (NAFLD-MS score). Liver Int. 2017; 37(10):1535-43.
17. Lee HW, Park SY, Kim SU, Jang JY, Park H, Kim JK, Lee CK, Chon YE, Han KH. Discrimination of Nonalcoholic Steatohepatitis Using Transient Elastography in Patients with Nonalcoholic Fatty Liver Disease. PLoS One. 2016; 11(6):e0157358.
18. The World Bank. Life expectancy at birth, total (years) - Thailand. https://data.worldbank.org/indicator/SP.DYN.LE00.IN?locations=TH. Accessed 31 Jan 2020.
19. Briggs A, Sculpher M, Claxton K. Decision Modelling for Health Economic Evaluation: OUP Oxford; 2006.
20. Limwattananon S. Handling uncertainty of the economic evaluation result: sensitivity analysis. J Med Assoc Thai. 2011; 91(6):59.
